# Tilted frames of reference have similar effects on the perception of gravitational vertical and the planning of vertical saccadic eye movements

**DOI:** 10.1007/s00221-015-4282-0

**Published:** 2015-04-29

**Authors:** Michael Morgan, Simon Grant, Dean Melmoth, Joshua A. Solomon

**Affiliations:** Max-Planck Institute for Metabolism, Cologne, Germany; City University London, London, UK

**Keywords:** Perception, Saccades, Gravity, Vertical, Frames of reference

## Abstract

We investigated the effects of a tilted reference frame (i.e., allocentric visual context) on the perception of the gravitational vertical and saccadic eye movements along a planned egocentric vertical path. Participants (*n* = 5) in a darkened room fixated a point in the center of a circle on an LCD display and decided which of two sequentially presented dots was closer to the unmarked ‘6 o’clock’ position on that circle (i.e., straight down toward their feet). The slope of their perceptual psychometric functions showed that participants were able to locate which dot was nearer the vertical with a precision of 1°–2°. For three of the participants, a square frame centered at fixation and tilted (in the roll direction) 5.6° from the vertical caused a strong perceptual bias, manifest as a shift in the psychometric function, in the direction of the traditional ‘rod-and-frame’ effect, without affecting precision. The other two participants showed negligible or no equivalent biases. The same subjects participated in the saccade version of the task, in which they were instructed to shift their gaze to the 6 o’clock position as soon as the central fixation point disappeared. The participants who showed perceptual biases showed biases of similar magnitude in their saccadic endpoints, with a strong correlation between perceptual and saccadic biases across all subjects. Tilting of the head 5.6° reduced both perceptual and saccadic biases in all but one observer, who developed a strong saccadic bias. Otherwise, the overall pattern and significant correlations between results remained the same. We conclude that our observers’ saccades-to-vertical were dominated by perceptual input, which outweighed any gravitational or head-centered input.

## Introduction

In the classic rod-and-frame paradigm introduced by Witkin and Asch (1948), a vertical rod inside a frame tilted in the fronto-parallel plane (roll direction) is seen as tilted in the opposite direction to the frame, implying that the subjective vertical has shifted in the same direction as the frame. This ‘rod-and-frame’ illusion (RFI) has received extensive investigation. There are large individual differences in the effect (Spinelli et al. 1995). Some subjects, termed ‘field dependent’ by Witkin and Asch, show large effects of the frame, while others show only small effects, or none at all. Other experiments, designed to test the influential perception–action model (Goodale and Milner 1992; Goodale et al. 1991), have sought to determine whether tilted visual reference frames affect action in the same way as they affect perception. At first sight, the results of these experiments have been contradictory. Dyde and Milner (2002) reported that a tilted reference influenced perception (by the method of adjustment) but not action (distance between thumb and forefinger when grasping the rods). However, Craje et al. (2008) found that the grip orientation used to grasp a rod was affected by a surrounding frame in the same way as an actual tilt of the rod from the vertical. Craje et al. argued that differences in task constraints may have contributed to the different findings (Smeets et al 2002). In the Dyde and Milner study, participants grasped the ends of the rod between their thumb and forefinger and were therefore dependent on the visual information regarding the *positions* of the ends of the rod, while in the Craje et al. study (so these authors argue), orientation of the rod was the key information. However, Dyde and Milner (2002) also report a perception–action dissociation for the RFI when the action was posting a card against a grating set in the center of a large tilted frame and conclude that the dissociation is *not* specific to grasping. Therefore, the circumstances in which the RFI affects behavior are not at present clear.

The effect of a visual reference frame has also been investigated with the Roelofs effect, in which the apparent position of a small central target is altered when it is presented inside a large frame, out of left–right alignment with the observer’s median plane. Contrary to its effect of perception, the frame has been reported as having no effect on manual pointing or saccades (review by Cardoso-Leite and Gorea 2010). However, the perceptual task in this case consisted of comparing the perceived target position with that of memorized targets previously presented at various positions relative to the median plane. As Cardoso-Leite and Gorea point out, a parsimonious explanation for the apparent dissociation is that the frame causes a shift in the perceived position of the subject’s median plane. This would shift the perceived position of a target previously encoded relative to the median plane, but would not affect an eye movement planned in egocentric coordinates. This example shows the importance of clear reasoning before taking a perceptual-motor dissociation as evidence for ‘two systems,’ an imperative we were aware of in designing our experiments.

The purpose of the experiments we report here was to investigate the effect of visual reference frames on saccadic eye movements. Specifically, we wished to determine whether the control mechanism for saccades made in egocentric coordinates is affected by tilted frames of reference in the same way that apparent spatial relationships are. To examine this question, we used a task in which participants were required to make a saccade to a virtual target, rather than explicit target. We call this the ‘saccade-to-vertical’ task (Barnett-Cowan and Harris 2008). Specifically, observers fixated a point inside a circle and when the fixation point disappeared, attempted to make a saccade to a point on the circle vertically below the fixation point (the ‘6 o’clock’ position). There was no explicit visual target, other than the circle. The results we describe show that participants could perform this task accurately. In principle, planning a ‘saccade-to-vertical’ could rely on several sources of information. One might be the *perceived* location of the 6 o’clock position provided by the visual context of the reference frame. Elsewhere, we have reported evidence that subjects can make accurate (oblique) saccades to a virtual target defined by a pointer presented in the context of a Poggendorff tilt illusion and that such eye movements are influenced by the visual context (Morgan and Melmoth 2011; Melmoth et al. in press). If visual information is mainly used to plan a vertical saccade, then the saccade should be affected by visual context in the same way as perception. Individual differences between ‘field-dependent’ and non-dependent subjects would also be reflected in their saccades-to-vertical. Another possibility is that the frame affects the perceived allocentric position of the target, but not the perceived egocentric position, in which case saccades should not be affected by the frame.

If not visual context, which sources of visual information do non-dependent subjects use? One possibility is that they are using a retinal coordinate system in which the vertical meridian is explicitly represented. Such a system would be vulnerable to head tilt and become less reliable than visual context. We predicted that head tilt would transform non-dependent individuals into field dependent. This was tested by combining the tilted and untilted frames with a 5.6° anti-clockwise head tilt, which is well beyond the range of compensatory cyclotorsion (Maxwell and Schor 2006).

To compare a frame’s effect on the saccade-to-vertical task with its effect on apparent spatial relationships, it was necessary to develop a perceptual test using the same stimulus. An obvious possibility would be to ask participants whether a briefly flashed dot was to the left or right of the 6 o’clock position (Tomassini et al. 2014). However, this ‘method of single stimuli’ is open to the critique that it confounds perceptual, decision and response biases (Morgan et al. 2012). A subject has only to respond in one of the two directions consistently when unsure of the correct answer to produce a bias in the psychometric function that is, in principle, indistinguishable from a genuine perceptual bias/illusion. This strategy need not necessarily be conscious. The reason for thinking that this may be important is that key results on context obtained with the method of single stimuli (Taya et al. 2009; Turi and Burr 2012) have not been confirmed using the two-alternative (2AFC) technique we describe here (Morgan 2013, 2014). The technique (technically ‘two-interval forced choice with a roving pedestal’) is to present two stimuli in temporal succession rather than one and to ask the subject to decide which of the two is *nearer* to the 6 o’clock position. By varying the actual location of *both* stimuli with a pedestal offset over a series of trials, it can be arranged that any genuine bias will affect the first and second stimulus equally and that the subject has no information on a given trial about which stimulus is actually closer to vertical. A decision bias of the kind ‘select the first stimulus if unsure’ will not mimic a genuine perceptual bias using this technique. A preliminary account of results obtained with this technique has been published elsewhere (Morgan et al. 2013).

In summary, we measured perceptual accuracy and context effects on the location of the apparent 6 o’clock position using a 2AFC method with a roving pedestal and saccade biases using ‘saccade-to-vertical.’ In both cases, three frames were used: (1) a control where the frame had zero tilt and two experimental conditions, in which the frame was tilted by (2) 5.6° in an anti-clockwise direction or (3) 5.6° in a clockwise direction. We also investigated the effect of a head tilt (5.6° anti-clockwise) under clockwise and anti-clockwise frame tilts, both for perceptual and for saccade tasks, with the hypothesis that tilting the participant’s head would make them more likely to use perceptual cues such as the frame and less likely to use gravity, thereby enhancing the frame effect.

## Methods

### Participants

The participants were the four authors, all highly experienced in psychophysical experiments, but not with the 6 o’clock task or with the RFE, and one naïve optometry student (ER). In addition, we used two further naïve observers (BM and JF) in the psychophysical test, but not with eye movements.

### Experimental setup and apparatus

 The experiments were carried out in a room with black window blinds and no source of illumination other than the screen and the dimly visible infrared light source for the Eyelink™ system used to record eye movements. Participants remained seated in front of the screen with their heads restrained by an Eyelink chin-and-forehead rest. In the head-tilted condition, the head rest was rotated in the roll direction by placing a wooden block under the right side, and the participant was more severely restrained by packing foam material between the head and sides of the rest. Stimuli were presented on a SONY Trinitron ™ monitor with resolution 1400 × 1050 pixels, viewed at 0.73 m so that 1 pixel (0.36 mm) subtended 0.03° of visual angle (DVA). Background luminance was 55 cd/m^2^, while luminance of the stimulus components was 130 cd/m^2^.

**Fig. 1:**
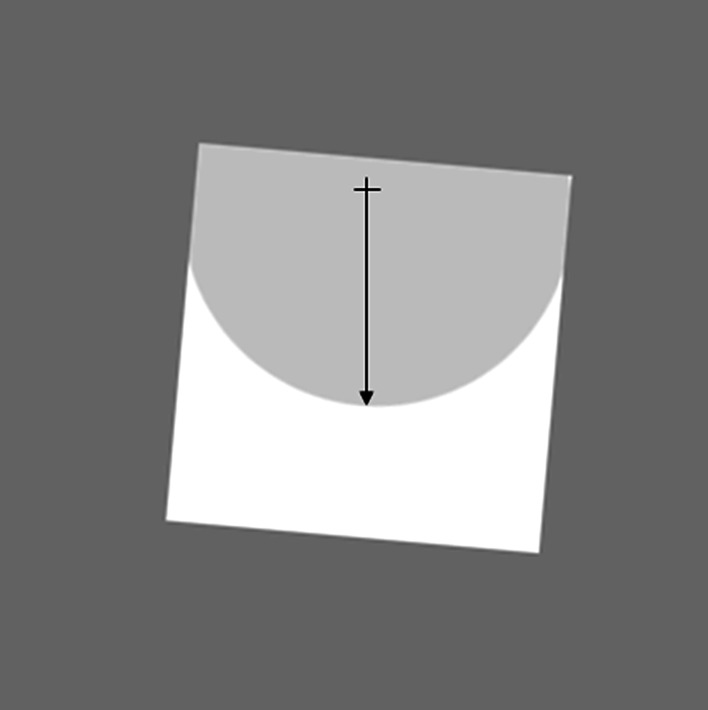
Schematic diagram of the display and frame as it was seen by the observer in the fronto-parallel plane under the clockwise-tilted frame condition. Participants fixated on the cross, and when the cross was extinguished made a vertical saccade (*arrow*) toward the estimated 6 o’clock position on the arc

Figure 1 shows a schematic view of the stimulus and frame as it appeared to the participants, with an arrow indicating the required direction of the saccade in the saccade-to-vertical. The fixation point (white on black background) was placed in the center of a disk (also white with luminance 52 cd/m^2^) with radius 7.5 DVA, visible to the subject as an arc within the frame. The actual position of the fixation point and circle within the frame was jittered over trials to avoid the subject using landmarks on the screen and stereotyped responding. The rest of the circle was occluded by the frame. The test dots for the perception task were placed at some point on the circle. Dimensions of the frame were 13.8 × 13.8 DVA. In different blocks of trials, the frame was tilted either 0, +5.6° (i.e., anti-clockwise) or −5.6°.

### Procedure

#### Perception task

**Fig. 2:**
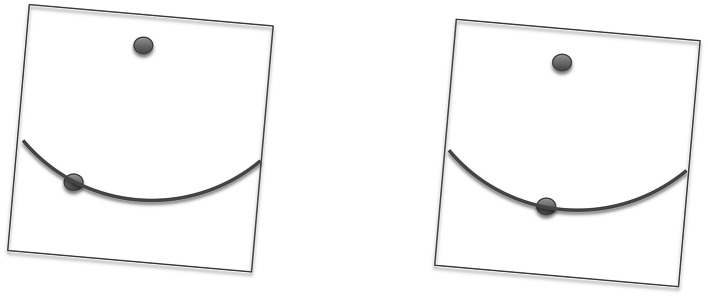
2AFC (temporal) with roving pedestal task for measuring the effects of frame tilt upon perception of the apparent vertical. The observer’s task was to decide whether the *dot pair* in the first (left) or second (right) interval is closer to the gravitational vertical. Both *dots* were given an offset from the vertical in the same direction (the pedestal offset) to which was added a variable offset in either directions for one of the stimuli (the target). In this case, the CW pedestal only is on the left; addition of the ACW cue on the right makes the two dots more aligned with the vertical

The stimulus configuration for the 2AFC task with roving pedestal is illustrated in Fig. 2. On each trial, the fixation point was presented for 0.5 s, followed by the first 2AFC interval of 1 s. During this interval, the fixation point remained visible and was joined by a second dot at some point on the lower circumference of the circle. We define the position of the test dot in polar coordinates with respect to the fixation point as (*r*, *θ*). This was followed after a brief blank interval by the second 2AFC interval, with a dot positioned on the circle at position (*r*, *θ*′). The subject’s task was to report whether *θ* or *θ*′ was closer to the subjective vertical. In other words, ‘was the first or second dot closer to the vertical with respect to the fixation point?’ To clarify what we meant by ‘the vertical,’ we told subjects that this was gravitational: at right angles to the floor, or equivalently, the shortest line from fixation point to the feet, which were placed directly under the table carrying the monitor. One of the probes, the *standard,* had a pedestal angle chosen from the set {−2°, 0, 2°} with respect to the vertical. The other, the *target*, had angle pedestal +*x*, where *x* was chosen from the list {−4°, −3°, −2°, −1°, 0, 1°, 2°, 3°, 4°}. The temporal order of target and standard was random. Over a series of 216 trials, every combination of standard and target was presented eight times in random order. In other words, the three pedestals were randomly interleaved so the subject could not know on any trial whether either of the dots was in a true vertical position. Nor did they know which dot was the reference. Thus, no response bias along the lines of ‘choose the reference if unsure’ could masquerade as a perceptual bias. On the other hand, with three psychometric functions (i.e., one for each pedestal), any bias due to the frame tilt can be decoded. The entire experiment was performed once with frame angle 0, once with frame angle −5.6° and once with frame angle +5.6°.

#### Eye movement task

Eye movements were monitored with an Eyelink 1000™ recorder. Each block of trials was preceded by a calibration and subsequent verification with a 9-dot display (standard Eyelink procedure) and then by a subsequent verification in which the participant moved his or her eyes around the four inside corners of the frame. This was then followed by a block of 18 trials, each of which began with the fixation point disappearing and the participant moving his/her gaze as rapidly as possibly to the 6 o’clock position on the circle, as previously defined for the perception task. A period of 0.5 s was allowed to make this saccade, after which the screen went blank. After an interval of 1.5 s, the fixation point reappeared and subjects moved their gaze back to the fixation point. After each block of 18 trials, the participant rested for at least 1 min, before starting a new block. At least three blocks of data were collected from each observer in each condition.

Saccade trajectories and landing positions were analyzed with a combination of Eyelink™ and custom software. The criterion for the end of the first (primary) saccade was that velocity fell below 30 DVA/s for a period of at least 0.020 s. No further saccades were included in the analysis.

### Psychophysical model

Within the context of signal detection theory (Green and Swets 1966), appearances of the standard and target can be described by normal distributions *S* and *T*, such that *S* ∼ *N*(*p* + *μ*, *σ*^2^/2) and *T* ∼ *N*(*p* + *t* + *μ*, *σ*^2^/2), where *σ*^2^ is the variance of the performance-limiting noise, *p* and *p* + *t* represent the physical tilts of standard and target, and *µ* represents any perceptual bias, such as may be induced by the frame angle *f*. All tilts are signed, such that negative values represent clockwise tilts. Given these definitions, the probability of choosing the standard in our comparison-of-comparisons task is given by.

1 $$\begin{aligned} \Pr \left( S \right) = & \Pr (\left| S \right| < \left| T \right|) \\ = & \Pr \left( {\frac{{S^{2} }}{{T^{2} }} < 1} \right). \end{aligned}$$

Note that *S*^2^/*T*^2^ is a random variable having a doubly non-central *F* distribution. Its denominator’s non-centrality parameter is 2(*p* + *μ* + *t*)^2^/*σ*^2^, its numerator’s non-centrality parameter is 2(*p* + *μ*)^2^/*σ*^2^, and both denominator and numerator have 1° of freedom.^1^

## Results

### Perceptual task

**Fig. 3:**
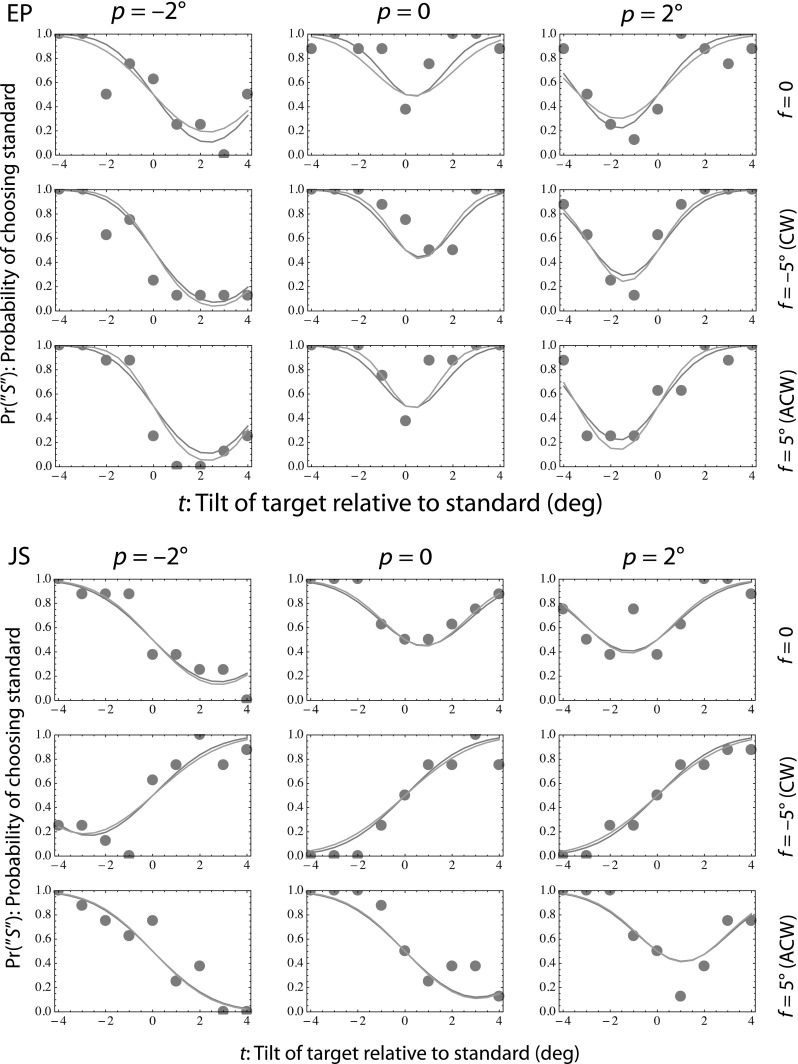
Figure shows individual psychometric functions of the perceptual test for two participants, one (JS,* bottom*) showing an effect of the frame and the other (EP,* top*) not. Each of the small panels shows the probability (*vertical axis*) of choosing the standard (having only the pedestal tilt) as being closer to the vertical, as a function of the tilt of the target relative to the standard (*horizontal axis*). In each row, the columns show results for different pedestal tilts and the rows show results for different frame tilts. The *orange curves* are six-parameter fits, in which the perceptual bias (*μ* and precision (1/1*σ*.*σ*)) was allowed to vary with each frame tilt. The *blue curves* (often overlapping the *orange*) are four-parameter fits, in which only bias (but not precision) was allowed to vary with each frame tilt. For further explanation, see text (color figure online)

**Fig. 4:**
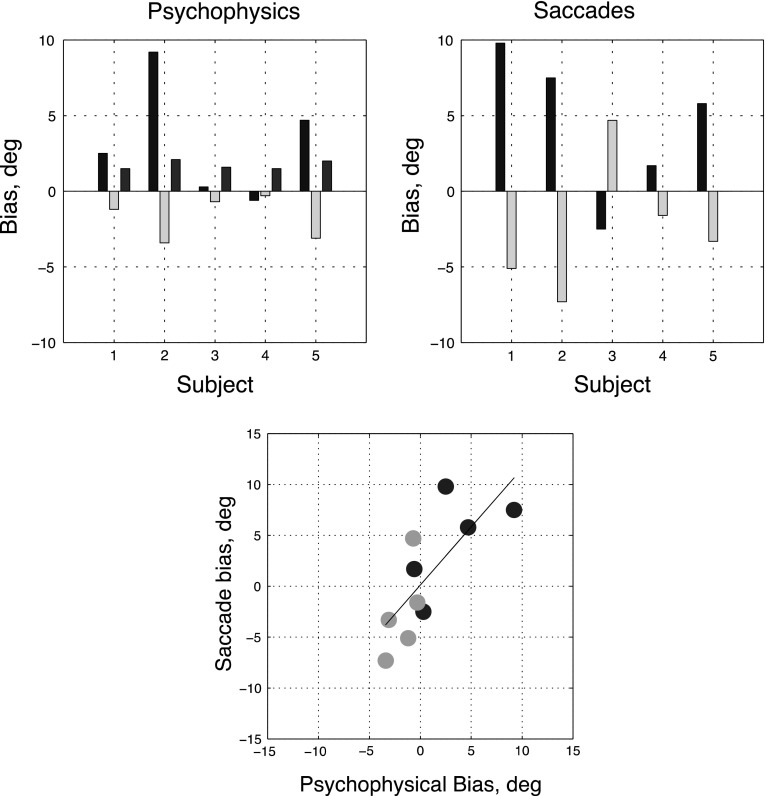
Relationship between individual subject biases in the perceptual and saccade-to-vertical tasks. The *top left panel* shows the effects of clockwise (*green bars*) and anti-clockwise (*blue bars*) frames upon the perception of gravitational vertical, inferred from the mean perceptual biases. Also shown are the standard deviations of the psychometric functions obtained by combining all frames (*brown bars*). The *top right panel* shows median saccadic endpoint displacement from the vertical using the same convention. The *bottom panel plots* the saccadic biases (*vertical axis*) against the psychophysical ones (*horizontal axis*) and the regression line through the data. The correlation between perceptual and saccade bias is 0.78 (*p* = .008). In order, the participants are MM, SG, DM, EP and JS (color figure online)

Psychometric functions for each combination of pedestal and frame tilt are shown for two representative participants in Fig. 3. Consider the simplest case: *p* = 0 and *f* = 0 (top row, second column). Here, the standard is always vertical and is seen as such. Tilts applied to the target move it away from the vertical, resulting in increased probabilities of choosing the standard (i.e., as appearing more vertical). Now consider *p* = −2° and *f* = 0. Positive tilts applied to the target bring it closer to the vertical than the standard, resulting in choice probabilities for the standard of $\Pr \left( {S} \right) < 0.5$. Tilting the frame by 5.6° in the anti-clockwise or clockwise directions resulted in participants MM, SG and JS always judging the standard to be more vertical than the target (i.e., with probability = 1) when it was tilted in the same direction as the frame. That is, their perception of gravitational vertical was determined by the frame tilt (‘field dependent’), whereas perceptions of the vertical for participants DM and EP were independent of the frame orientation (Fig. 4).

Psychometric data were maximum likelihood fit to Eq. (1) twice. In the six-parameter fit (orange curves), the performance-limiting noise was allowed to vary with the frame angle; in the four-parameter fit (blue curves), it was not. Negative values of *μ* represent clockwise biases in the perception of vertically oriented stimuli, and positive biases represent the reverse. For two subjects (DM, EP), frame tilt did not affect bias: A two-parameter fit to the data from each of these subjects was not significantly inferior to the four-parameter fit (Hoel et al. 1971). For the remaining three participants illustrated in Fig. 4 (MM, SG and JS), perceptual biases were in the direction opposite to the frame tilt, as in the classical rod-and-frame effect. In addition, two further naïve subjects were run (not shown in the figure because we have no saccade data for them). Both showed a frame effect. For BM, the net frame effect, ${{\left( {\mu_{{{\text{CW}}{\kern 1pt} {\text{frame}}}} - \mu_{{{\text{ACW}}{\kern 1pt} {\text{frame}}}} } \right)} \mathord{\left/ {\vphantom {{\left( {\mu_{{{\text{CW}}{\kern 1pt} {\text{frame}}}} - \mu_{{{\text{ACW}}{\kern 1pt} {\text{frame}}}} } \right)} 2}} \right. \kern-0pt} 2}$, was −1.7°, and for JF, it was −2.15°. The *σ* values for their psychometric functions were 1.5° and 2.5°, respectively. Thus, out of total of seven participants, five showed a frame effect and two did not.

As previously discussed, in the classic rod-and-frame effect, a vertical rod inside a tilted frame is seen as tilted in the *opposite* direction to the frame. This implies that the perception of gravitational vertical has shifted in the *same* direction as the frame. Values for this latter shift, inferred from (i.e., opposite to) the perceptual biases (in degrees of roll), are shown in Fig. 4. Values for precision (also in degrees of roll) are also provided. Both values were derived from the four-parameter fits. For clarity, confidence intervals have been omitted from this figure. They are illustrated later in Fig. 6.

### Saccade-to-vertical task

**Fig. 5:**
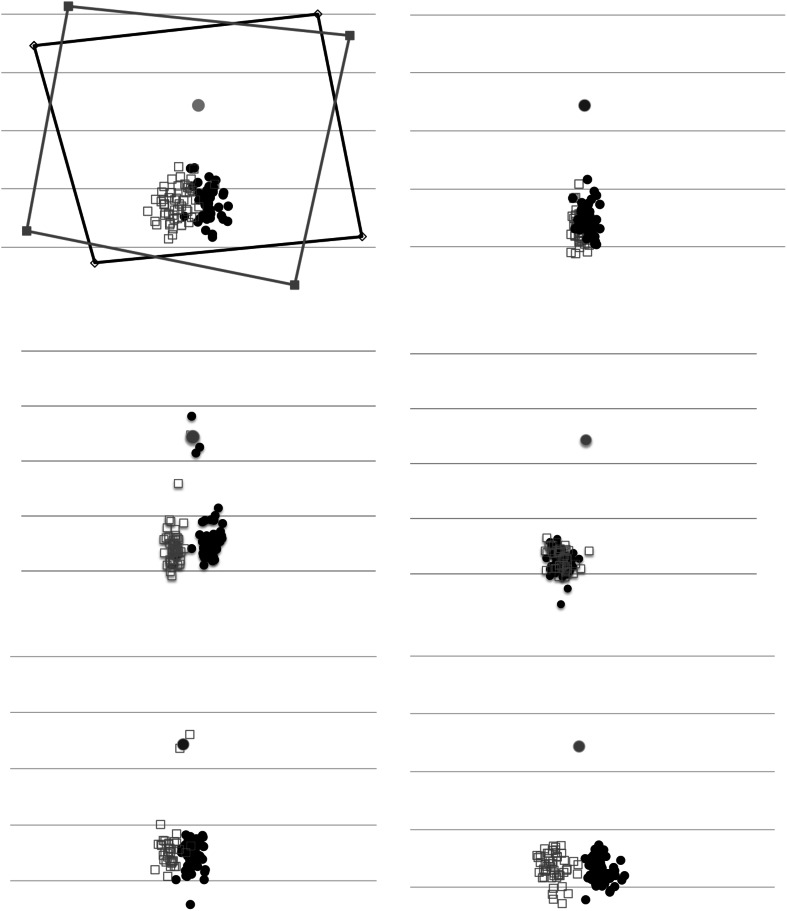
*Blue symbol* in the center shows the actual position of the fixation point before the saccade. *Red symbols* show individual saccade endpoints with a CW-tilted frame; *black symbols* with an anti-clockwise tilted frame. Panels, reading from* left to right* and* up to down*, show results for MM, EP, SG, DM, JS and JS (tilted head). The frames in the *top left panel* show the endpoints of saccades directed to the four comers of the tilted frame(s) (color figure online)

Representative scatter plots of the saccade endpoints for four subjects, two showing a clear effect of the frame and two showing a smaller or no effect, are shown in Fig. 5.

The three participants on the left (MM, SG, JS) show a large effect of the frame. Participant EP (top right) shows a smaller effect, with overlap between the two distributions, and participant DM (middle right) showed no effect of frame. The bottom right panel shows results for JS in the head-tilted condition.

To minimize the influence of a few obvious outliers (see Fig. 5), we summarize each participant’s saccadic endpoints using the median value in each task condition. These median values are shown in the right-hand top panel of Fig. 4. For four of the participants (1, 2, 4 and 5), saccadic endpoints were shifted away from the true (i.e., gravitationally defined) 6 o’clock position in the same direction as tilted frame. One participant (three) showed an effect in the opposite direction.

Figure 4 also shows (bottom panel) the relationship between estimates of gravitational vertical inferred from the perceptual and saccadic tasks. It is clear from the significant positive correlation (*ρ* = 0.78, *p* = 0.0077) that the same participants who showed a significant perceptual effect of the frame also showed an effect on saccadic endpoints and that the effects were of comparable magnitude. Indeed, the three participants (1, 2 and 5) showing the largest perceptual effect also showed the largest effects on saccades.

### Results with tilted head

#### Perceptual with non-tilted frame

**Fig. 6:**
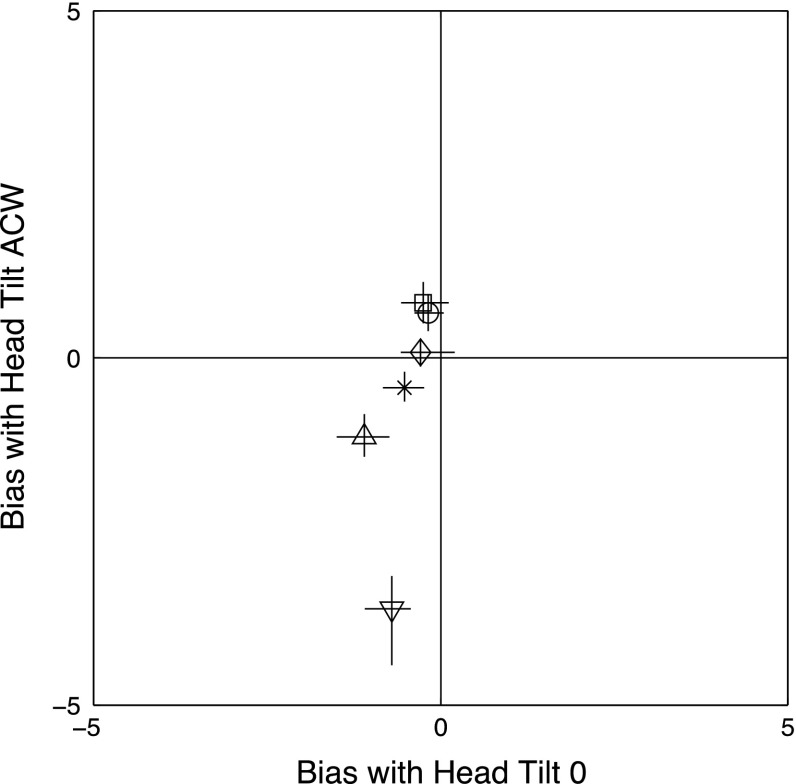
*Each symbol* in the figure shows bias in the perception of vertical (i.e., −*μ*) for one participant in the untilted head condition and with head-tilted ACW. All data were collected with an untilted frame. The symbols for the five participants identified in Fig. 4 are, in order: *circle*, *square*, *x*, *diamond*, *downward pointing triangle*. To this are added the results for a sixth participant, identified by an *upward-pointing triangle*. The *error bars* show 95% confidence intervals, calculated by fitting nonparametric bootstrap samples (*n* = 160) with the approximate formula of Morgan et al. (2013). Sufficient computing power was not available for fitting these samples with the exact formula

If tilting of the head produces a perceptual bias, this should be seen in the condition where the frame was upright, i.e., not tilted. Figure 6 shows the results in this condition, comparing the tilted head and untilted head conditions.

Most of the subjects show small biases that are not significantly greater in the head tilted than in the head-untilted condition. One participant (5 in Fig. 4) shows a large bias in the head-tilted condition, and the additional participant (6) has a smaller bias in the same direction. The reason for these biases is not known.

#### With tilted frames

**Fig. 7:**
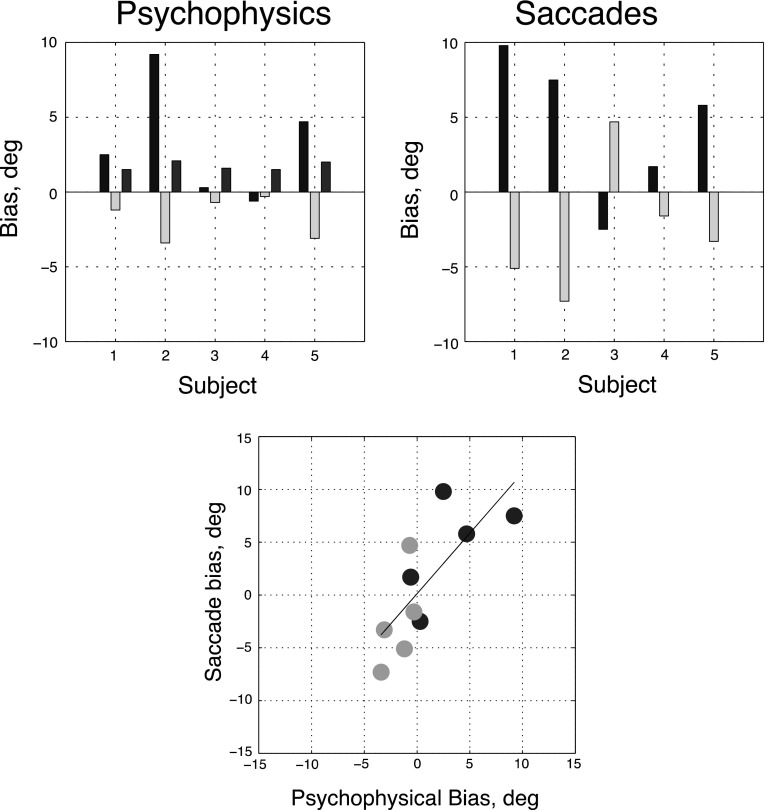
Relationship between individual subject biases in the perceptual and saccade-to-vertical tasks with the head tilted relative to the body. Conventions as in Fig. 4. The correlation between perceptual and saccade bias is 0.83 (*p* = .003)

**Fig. 8:**
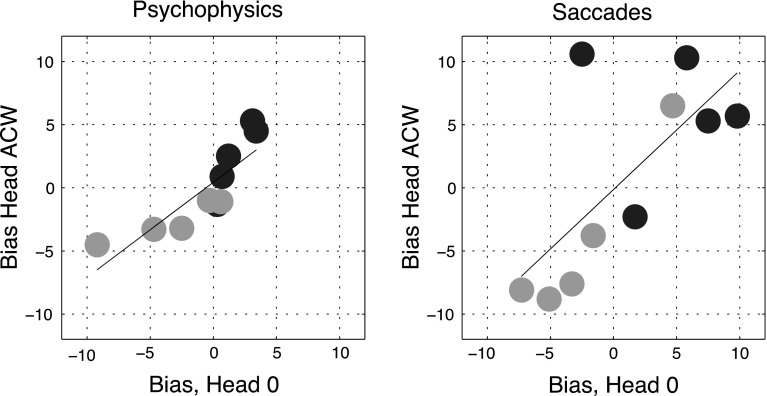
Figure shows the relationship between biases with an upright head (head 0) and a tilted head (head ACW). Each *blue* and *green circle* represents the biases of a single participant in the ACW- and CW-tilted frame conditions, respectively. Results for the psychophysical task are shown in the *left-hand panel* and those for the saccade task on the right. The correlation for the psychophysical task is 0.87 (*p* = .0011); correlation for saccades is 0.71 (*p* = .023). Note the clear outlier (participant 3) in the clockwise frame saccade task, with a bias in the head ACW condition of about −10° and of nearly zero in the head 0 condition (color figure online)

Figure 7 shows that biases in the perception of gravitational vertical with the tilted head were broadly similar to those with the head upright. The distinction between ‘frame-dependent’ and independent participants was maintained. Indeed, the correlation between these biases in the head-upright and head-tilted conditions (see Fig. 8) was 0.87 (*p* = 0.001) across all five subjects. Saccade biases were also broadly similar between the two conditions of head tilt with a correlation of 0.71 (*p* = 0.02). There was, however, one interesting exception. Participant 3, who was non-dependent and who had shown a reversed saccade bias with the upright head (Fig. 4), showed a large negative saccade bias with a tilted head (Fig. 7), under both conditions of frame tilt. The same participant showed little perceptual bias and is thus an outlier in the lower panel of Fig. 7, despite which, the overall correlation between perceptual and saccade biases across subjects with head tilt remained strongly positive (*ρ* = 0.73, *p* = 0.016).

The results for this participant are consistent with his planning saccades in a retinally centered or a head-centered coordinate system. An anti-clockwise head rotation, unless countered by cyclotorsion, would shift the retinal vertical meridian anti-clockwise relative to the CRT, and it would displace the saccade-to-vertical anti-clockwise relative to the outside world, just as it would in a frame-dependent participant. It is most unlikely that the 5.6° head tilt employed could have been fully countered by torsional re-alignment of the eyes in any of our participants, since such binocular cyclorotations are reported to be limited to only ~1° in normal adults (Maxwell and Schor 2006). Thus, an anti-clockwise *head* rotation of 5.6° in the roll direction in a retinally centered participant should have the same directional effect as an anti-clockwise *frame* rotation in a frame-dependent subject, namely a positive bias, which is what we observe for his saccades.

The other non-dependent participant (four), on the other hand, shows little saccade-to-vertical bias in the head-tilted condition. This near-veridical performance suggests that the subject was using a body—or gravitationally defined frame of reference to plan the saccade. Note that such alternative references must also have been used by participant three in the head-upright perceptual task, because he shows very little bias associated with the visual context of the frame orientation. This is the only clear dissociation between perception and saccade planning that we found in our experiments.

## Discussion

Our finding that a tilted frame of reference can displace vertical eye movements confirms that of Barnett-Cowan and Harris (2008) but with considerably smaller tilts. Barnett-Cowan and Harris placed observers in a room that could be tilted relative to the vertical and asked participants to make repetitive vertical eye movements, either with respect to their head orientation or with respect to gravity. Both kinds of saccade were shifted from the vertical by tilting the room. In contrast to our procedure, there was no explicit target for the saccade, whereas we had a visible arc to which the participants were asked to make a saccade. A further difference is that we tilted only a single rectangular frame, rather than the whole room. Finally, our (5.6°) tilts were considerably smaller than those used by Barnett-Cowan and Harris (60° and 120°). It would be interesting to see whether the performances of our ‘frame-independent’ observers would be immune to an extremely tilted, immersive environment like this. If not, we would have to conclude that our ‘frame-independent’ observers merely gave a relatively low weight to our slightly tilted rectangle. In other words, the difference between them and ‘frame-dependent’ observers could be quantitative rather than qualitative.

Spering and Carrasco (2015) have recently reviewed dissociations between visual perception and eye movements, for example, greater sensitivity of smooth pursuit than perception to threshold changes in target velocity (Tavassoli and Ringach 2010). Our findings do not show such a dissociation in the case of a tilted frame, which had similar effects on saccades and a perceptual task. Indeed, individual differences between participants were reflected in both their perceptual and motor responses. The only possible exception is the fact that EM showed no frame effect in the perceptual task (with head upright) and a small but consistent effect on saccades. This is just a hint that the saccade task may be more sensitive, but statistical reasons would need to be ruled out before this could be accepted as a real difference in relative sensitivity.

Previous investigations have revealed inconsistent effects of a tilted reference frame upon manual behavior, depending on the task (Dyde and Milner 2002; Craje et al. 2008). Our purpose was to see whether a tilted reference frame would affect eye movements in a ‘saccade-to-vertical’ task. It did. From this, we conclude that planning of a saccadic eye movement can be influenced by visual context, as also appears to be the case for the Poggendorff illusion (Morgan and Melmoth 2011; Melmoth et al. in press). We have also shown that individual differences, previously reported for the perceptual frame effect (Spinelli et al. 1995; Witkin and Asch 1948), are found in the saccade-to-vertical task too and that participants who have little or no perceptual bias have either no saccade bias or one in the ‘wrong’ direction. We also find that both perceptual and saccade biases due to a visual reference frame occur in a head-tilted condition, in which it might be anticipated that the subject’s egocentric encoding of gravitational vertical (i.e., straight down toward their feet) would be less affected by the visual context.

We initially thought it quite possible that saccadic eye movements might have a different balance of inputs from gravitational, visual context and body-frame cues from perception. The ability of subjects to make accurate second saccades in a double-step paradigm in the dark (Becker and Jurgens 1979) suggests that eye movements can be programmed in a body-centered coordinate frame. However, our results show that ‘frame-dependent’ subjects in the perceptual test are also ‘frame-dependent’ in the ‘saccade-to-vertical’ test, and regardless of their head position, arguing that they do not use a body—or other gravitational-centered reference frame for their saccades under either of these task conditions.

## Footnotes

1 Whereas the formula given on page 202 of Morgan et al. (2013) is a close approximation, Eq. (1) is exact.
